# Literary Notes

**Published:** 1901-01

**Authors:** 


### LITERARY NOTES.

Messrs. Battle & Co., of St. Louis, have issued chart No. 2 of the series illustrating fractures of the large bones. The subject of this number is oblique fracture of the shaft of the tibia. It is well drawn and the muscles in color make the work effective.

---

A contribution to the therapeutics of Pepto-Mangan, Gude, is the title of a paper by Dr. Ludwig Pohl, city physician, Vienna, Austria. It was originally published in the *Aerztlicher Central Anzeiger*, Vienna, and is reprinted and sent out by Messrs. M. J. Breitenbach Co., agents for the American continent of Pepto-Mangan, Gude. Dr. Pohl gives reasons in detail for the benefits derived from this preparation where iron is indicated in an easily absorbable form. He regards it as an excellent preparation and one that bids fair to occupy a permanent place in the materia medica.

---

Messrs. D. Appleton & Co. announce that they have in press A Text-book of Gynecology, edited by Charles A. L. Reed, A.M.,

M.D., president of the American Medical Association; gynecologist and clinical lecturer on diseases of women in the Cincinnati Hospital, etc. This work is an exposition of the practice of medicine, in its broadest sense, as applied to diseases peculiar to women.

It will shortly be issued in a single 8vo volume of nearly a 1,000 pages, illustrated with about 400 original drawings by Mr. Roy J. Hopkins. It will be sold at $5.00 in cloth, and at $5.50 in Appletons' Medical Library style.

---

THE *Western Reserve University News Letter* is the title of a leaflet issued weekly by the Western University, at Cleveland, O. Its object is to give the public authentic notes regarding the University. The subscription price is 25 cents a year, but it will be sent as an exchange to any editor who may express a desire to receive it. It is a novel idea and will undoubtedly prove beneficial to the University.

